# Do Acute Illness Perceptions Moderate the Association of Pre-Collision Welfare Benefits and Later Neck Pain or Disability Following Whiplash Trauma? A Prospective Multicentre Cohort Study

**DOI:** 10.3390/jcm13237072

**Published:** 2024-11-22

**Authors:** Tina B. W. Carstensen, Sophie L. Ravn, Tonny E. Andersen, Solbjørg M. M. Sæther, Eva Ørnbøl, Kaare B. Wellnitz, Helge Kasch, Lisbeth Frostholm

**Affiliations:** 1 Department of Functional Disorders, Aarhus University Hospital, 8200 Aarhus, Denmark; eva.oernboel@aarhus.rm.dk (E.Ø.); lisbeth.frostholm@aarhus.rm.dk (L.F.); 2 Department of Clinical Medicine, Aarhus University, 8200 Aarhus, Denmark; helgkasc@rm.dk; 3 Specialized Hospital for Polio and Accident Victims, 2610 Roedovre, Denmark; slravn@health.sdu.dk; 4 Department of Psychology, University of Southern Denmark, 5230 Odense, Denmark; tandersen@health.sdu.dk; 5 Department of Health Promotion, Norwegian Institute of Public Health, 5015 Bergen, Norway; makalani@myrtveit.com; 6 Department of Neurology, Aarhus University Hospital, 8200 Aarhus, Denmark

**Keywords:** whiplash injuries, health, knowledge, attitudes, practice, social risk factors, social welfare, sick leave, unemployment, health status, registries, questionnaires, multivariate analysis

## Abstract

**Objectives**: Whiplash trauma is a worldwide significant public health issue, with post-collision chronic pain and physical and mental disability; the prevalence of whiplash trauma in the Japanese general population is estimated at 1.2% and in the Danish general population the whiplash condition has been reported to be 2.9%. Pre-collision welfare benefits and illness perceptions have been found to predict poor recovery after whiplash trauma. In this study, we examined whether illness perceptions measured shortly post-collision moderated the effect of welfare benefits five years before the collision on neck pain and neck-related disability one-year post-collision. **Methods**: Patients consulting emergency rooms or general practices with neck pain after acute whiplash trauma were invited to complete questionnaires during the week after the collision and at three and 12-months post-collision. Further, we obtained register data on the number of weeks on three types of welfare benefits (sick leave benefits, unemployment benefits, and social assistance benefits) for a five-year period before the collision. Multiple logistic regression was applied. **Results**: 740 patients were included. We did not find a significant moderating effect of illness perceptions on the association between pre-collision welfare benefits and chronic neck pain and related disability. However, there was a trend towards illness perceptions at baseline and at the three-month follow-up having a moderating effect on the relationship between long-term sick leave and neck pain one year after the whiplash collision. **Conclusions:** Regarding long-term sick leave, we might have overlooked a substantial moderating effect due to methodological matters and recommend a replication of this study on a larger sample, also focusing on other recovery outcomes.

## 1. Introduction

People experiencing whiplash trauma commonly report symptoms like headache, neck pain, shoulder pain, and related disability [1]. While approximately 50% recover within three to six months, up to 50% continue to report symptoms [2]. When symptoms persist for more than six months, the condition is often termed chronic whiplash associated disorders (WAD) [3]. WAD is a worldwide significant public health issue with post-collision chronic pain and physical and mental disability [4,5]; the prevalence of WAD in the Japanese general population approximates 1.2% (1.3% in male and 1.0% in female) [6], and in the Danish general population, whiplash conditions are reported in 2.9% (2.1% in male and 3.7% in female) [7]. The factors leading to chronic WAD are likely both multifactorial and biopsychosocial [8,9] and seem also to be partially associated with pre-collision factors such as pain, poor general health, and a high use of health care [10,11,12]. While some types of welfare benefits have not been associated with WAD, such as pre-collision unemployment benefit [12,13], pre-collision social assistance benefit [12,14], and pre-collision disability pension [13], some studies have found an association with pre-collision sick leave benefits [12,13]. In a previous study based on the data used in this study, we found that receiving sick leave benefits (for >12 weeks during the five-year period pre-collision) was strongly associated with poor recovery post-collision [12]. Similarly, Myrtveit et al. [13] also found that pre-collision sick leave was associated with an increased risk of developing WAD. This might indicate that pre-existing vulnerabilities play a role in poor recovery in some patients.

The multifactorial nature of WAD can be understood in the light of the common-sense model of illness. According to this model, a person encountering a health threat like a whiplash trauma will develop perceptions of that health threat, which in turn regulate how one acts and responds to it [15]. Patients with the same illness will vary in terms of their perceptions of it, which may be important in understanding the distinct illness trajectories. Illness perceptions seem to play a role in outcome across a range of medical conditions such as cancer, inflammatory bowel disease, medically unexplained symptoms, rheumatoid arthritis, and irritable bowel syndrome [16,17,18,19,20,21], and a systematic review has shown associations between illness perceptions and work participation in patients with various complaints and illnesses [22]. Previously, we found that people exposed to acute whiplash trauma were more likely to report future neck pain and reduced working ability if they had negative illness perceptions at baseline and three months post-collision, and that this association increased from baseline to three months post-collision [23]. Hence, illness perceptions may be an important factor in understanding post-whiplash recovery. Despite this, no prior studies to our knowledge have investigated the interaction of illness perception and welfare benefits on recovery following acute whiplash trauma. Therefore, we aimed to examine whether whiplash-related illness perceptions measured immediately after the accident and three months later moderated the association between receiving three types of welfare benefits, pre-collision and neck pain intensity, and neck-related disability one year post-collision.

Studies investigating pre-collision unemployment benefits and social assistance benefits found no association with poor recovery [12,13,14], but pre-collision sick leave was found to be associated with poor recovery [12,13]. Therefore, we presume that different mechanisms within the three welfare benefits may influence later illness perceptions and later recovery or non-recovery. For social assistance and unemployment benefits, the effect of these welfare benefits might be primarily due to social exclusion, which, although not directly related to illness, still imposes life challenges with life baggage that can hinder recovery from whiplash trauma. However, this is not related directly to illness and therefore is probably not moderated by illness perceptions. In the case of sick leave, social exclusion may also play a role, but its impact is likely to be more pronounced since it directly relates to sickness, potentially shaping illness perceptions regarding the whiplash trauma itself. Therefore, we hypothesised that, in the case of sick leave, there may be a moderating effect of illness perceptions on the association between sick leave and poor outcome, but we did not expect that the same was true when it came to welfare benefits that are not related to sickness, i.e., social assistance and unemployment benefit. We expected that the potential moderating effect of illness perceptions increased from baseline to three months post-collision.

## 2. Materials and Methods

### 2.1. Study Design and Population

This study used a prospective multicentre cohort study design. Patients consulting emergency units or general practitioners with acute neck pain after car collisions were invited to participate in this multicentre study conducted by the Danish Pain Research Centre, Aarhus University Hospital, Aarhus, Denmark and the Back Research Centre, Odense University Hospital, Ringe, Denmark. The uptake area covered 1.7 million inhabitants in 2001. Patients from April 2001 to June 2003 were included in the study [12,23,24,25].

Potential participants were informed about the study and its design in a written invitation. Inclusion criteria were patients aged 18–70 years who had experienced neck pain within 72 h after being exposed to a rear-end or side-impact car collision. All patients were recruited within the first ten days of the collision, when they completed the baseline questionnaire. Patients were excluded if they could not be examined within ten days of the car accident, had fractures or dislocations of the cervical spine, had amnesia or unconsciousness in relation to the accident, had injuries other than the whiplash trauma, had no symptoms, had a significant pre-collision physical or psychiatric disorder, had self-reported average neck pain during the preceding six months exceeding five on a box scale of 0–10, or had alcohol or drug abuse issues.

The current study consists of secondary analyses performed on the entire cohort. Within the framework of this study, two randomised controlled trials (RCT) were performed. The RCTs only included a subgroup of the overall sample. The first RCT included patients with relatively severe initial symptoms who were randomised into three treatment groups: (1) immobilisation of the cervical spine in a semi-rigid collar, (2) advised to act as usual (no active treatment), and (3) active mobilisation. The second RCT included patients with less severe initial complaints who were randomised into two treatment groups: (1) a personally communicated patient education and (2) patient education using a pamphlet. No statistically significant differences were observed across the treatment groups in either RCT. For a detailed description of the RCT studies, please see Kongsted et al. [24,25].

### 2.2. Measures

Data used in the present study are a combination of self-reported questionnaire data and register data. Data comprise explanatory variables (three types of welfare benefits during the five years pre-collision), hypothesised moderators (illness perceptions at inclusion and at the three-month follow-up), outcome measures (neck pain and neck-related disability at the one-year follow-up), and covariates identified with directed acyclic graphs (DAGs) [26]. For details, please see below.

#### 2.2.1. Explanatory Variables

The Danish Register for Evaluation of Marginalization (DREAM) includes all individuals with a Danish social security number who have received social benefits or any other welfare payments since July 1991 [27]. DREAM contains weekly information on welfare payments comprising sick leave benefit, disability pension, unemployment benefit, maternity leave pay, state education fund grants, and so forth. A welfare payment is registered in DREAM for a whole week if the individual has received welfare benefit for at least one day during that week. Only sick leave periods exceeding two weeks are included in DREAM as the first two weeks are paid by the employer. If sick leave lasts for more than two consecutive weeks, the first two weeks are also counted.

In the present study, we included three types of welfare benefits as explanatory variables, as categorised by Carstensen et al. [12]; (1) social assistance benefits, (2) sick leave benefits, and (3) unemployment benefits. All three are counted as the number of weeks of welfare benefits received during the five years preceding the accident. The distribution of weeks for all three welfare payments were extremely right skewed. Subsequently, we grouped the observations based on normative criteria (see below).

Sick leave benefits covered individuals with or without employment, who were full-time or part-time ill, and individuals in activation programs or flexible jobs who were currently ill. Sick leave benefit was categorised according to its duration: (1) no sick leave benefit, (2) 1–12 weeks, and (3) more than 12 weeks. In Denmark, at the time of data collection, individuals could receive sick leave benefit for up to two years, and if one was still sick and unable to work after that period, the person got transferred to a permanent health-related benefit or social assistance. The period of 12 weeks was firstly based on the definition of chronic illness according to the World Health Organisation (WHO), which is the course of an illness lasting more than three months, with the intention of capturing both long-term and short-term sick leave. Secondly, it was based on previous research establishing long-term sick leave as being unable to resume work within three months of sick leave [28].

Unemployment benefits covered unemployed individuals (full-time or part-time, during vacation or activation, citizens on social assistance deemed able to work but unemployed), and persons receiving vocational rehabilitation benefits since they are in the period rehabilitated to the work force but unemployed. Unemployment benefit was categorised according to its duration: (1) no unemployment, (2) 1–52 weeks, and (3) more than 52 weeks. In Denmark during 2001–2003 it was not possible to receive unemployment benefit for more than four years, and individuals remaining unemployed after that period were transferred to social assistance, which entails a lower payment. The cut off at 52 weeks was based on the Organisation for Economic Co-operation and Development (OECD)’s definition of long-term unemployment as that involving people being out of work and looking for work for 12 months or more.

Social assistance benefits covered individuals who were unable to support themselves and thereby received social assistance benefit. This variable included social assistance, disability pension, and flexible jobs (a job with a low number of working hours supplemented by income from the government). Excluded from this variable were citizens on social assistance deemed ready to work, who were only receiving social assistance because of their unemployment, but who were otherwise not entitled to unemployment benefit. This group fell within the unemployment variable. Social assistance was categorised according to its duration: (1) no social assistance, versus (2) one week or more. This variable was categorised into only two groups as these benefits are typically long-term or given to marginalised groups of people (e.g., homeless people), so this level of severity is included in the dichotomisation of the variable.

#### 2.2.2. Hypothesised Moderators

The hypothesised moderators were illness perceptions formed in the acute phase (within the first ten days post-collision) and in the sub-acute phase (three months post-collision). Illness perceptions were measured using the Illness Perception Questionnaire Revised (IPQ-R), which is a theoretically derived measure that provides a quantitative measure of the components of the common sense model of illness [29]. The IPQ-R used in this study was a Danish version condensed and adapted for use in populations without a well-defined illness [30]. The Danish IPQ-R contained 20 questions concerning the patients’ perceptions of illness in subscales of identity, cause, timeline, consequences, perceived control, and emotional representations. The items measuring emotional representations were extended to include helplessness and hopelessness. The response options were true, mainly correct, mainly false, and false. Participants were also given the option to indicate “cannot answer”. Through principal components analysis it has previously been shown that this Danish version of the IPQ-R has a comparable dimensional structure to the original IPQ-R [30].

In this study, only the following illness perception subscales were included: emotional representations (5 items), consequences (5 items), and timeline perspectives (3 items). This was based on previous findings indicating that these illness perception subscales are more strongly associated with negative outcomes [21,30,31]. Examples of the statements posed were as follows: “My symptoms following the accident are signs of a prolonged condition” (timeline perspective), “My symptoms following the accident make me feel hopeless” (emotional representations), and “My symptoms following the accident have major consequences on my life” (consequences).

The sum scores of the included illness perception subscales were right-skewed, and the three variables were highly correlated (correlation range at baseline: 0.34–0.58, correlation range at 3-month follow-up: 0.57–0.71). As the three subscales had a different number of items we divided each sum score of the subscales by the number of items and then calculated an overall mean across the three subscales at baseline and at the 3-month follow-up.

#### 2.2.3. Outcome Measures

##### Neck Pain Intensity

At the one-year follow up, the participants rated their neck pain intensity during the preceding week on an 11-point box scale (0 = no pain, 10 = worst possible pain) [32]. Participants were instructed: “This is your assessment of your average neck pain during the last week, please tick it off even if you have no pain”. This variable can be considered a categorical variable or a continuous variable. The pain score was dichotomised on the basis of the work by Collins et al. [32], and is in line with already published articles from this multicentre study [10,12,23,24,25]. Scores from 0 to 3 were considered as “minimal pain” and scores from 4 to 10 were considered as “considerable pain”.

##### Neck-Related Disability

Neck-related disability was measured at the one-year follow-up using the 15-item Copenhagen Neck Functional Disability Scale (0 = no neck disability, 30 = extremely disabled) [33]. The scale includes questions regarding interference of sleep, daily activities, lifting, reading concentration, leisure time activities, and questions of a psychosocial nature all related to neck pain. Questions were answered with yes (0 points), occasionally (1 point), and no (2 points). The neck-related disability scale has been validated in other spinal pain populations [33]. The variable was included in the moderation analyses as a categorical variable (0/1) with disability scores from 0 to 6 defined as “minimal” based on previously suggested categories [24,25].

#### 2.2.4. Covariates

The selection of confounding variables was conducted in two steps: (1) identifying possible variables from the literature and (2) using DAGs constructed in the browser based program DAGitty version 3.0 [26], where a comprehensive list of potential confounders was entered. Based on a network of associations, DAGs provided the minimal number of confounding variables needed for generating the unconfounded direct and moderated effect estimates. The number of variables entered into the DAGs was 16. DAGs were established with guidance from an expert group containing statisticians, psychologists, and a clinical specialist. The final list of covariates to control for included sex, age, and education, all obtained at baseline within the first ten days post-collision through self-report. The covariates were included in all moderation models. Age was included as a continuous variable, sex as a binary variable, and level of education in five groups (‘unskilled’, ‘skilled’, ‘further education ≤ 4 years’, ‘further education > 4 years’, ‘other’).

### 2.3. Statistical Analyses 

#### 2.3.1. Descriptive Statistics

Descriptive statistics (count, percentages, mean, and standard deviation) were used to display socio-demographic variables, welfare benefits, illness perceptions, and outcomes in the study sample.

#### 2.3.2. Multiple Logistic Regression Analyses

**Figure 1:**
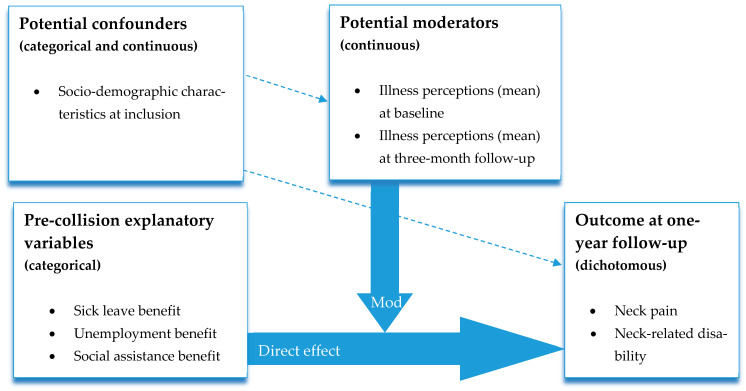
Graph of hypothesised moderator analysis. Mod = moderator/interaction effect. The dashed arrows represent possible effects that could induce confounding.

We fitted twelve models; one for each combination of the two outcomes (neck pain intensity, neck-related disability), the two moderators (mean illness perceptions at baseline, mean illness perceptions at the three-month follow-up), and the three explanatory variables (social assistance benefits, sick leave benefits, unemployment benefits in the five years preceding the accident). The reference group comprised unskilled males, 18 years of age at inclusion, receiving no weeks of social assistance, sick leave, or unemployment benefit within a five-year period before the collision and with a sample mean level of 0 on illness perceptions. All models were adjusted for sex, age at inclusion, and education level. The primary focus was the interaction between illness perceptions and welfare benefits (Figure 1). Results from the multiple logistic regression analysis are presented as odds ratios (OR) with 95% confidence intervals (CI). In accordance with Zieliński et al., we adhere to intervals for small (1.44), medium (2.48), and large (4.27) OR [34]. A moderation effect was considered to be present if the confidence interval of the interaction effect did not contain 1. Note that the measure of illness perceptions was a mean of three scales, meaning that a change of 1 in the moderator variable means a change of 1 on each of the three illness perception subscales.

To avoid over-fitting the models, we set the limit on the number of explanatory parameters as 10 cases available for each parameter [35,36], which allowed 16 parameters in the models containing illness perceptions at baseline, and 15 parameters in the models containing illness perceptions at the three-month follow-up. The twelve models included 11–12 parameters. We examined the linearity between the independent variables and the logit-transformed dependent variable using visualisations including scatter plots to identify potential non-linearities [36]. We tested with natural cubic splines and concluded that two continuous variables, age and illness perceptions, could be entered as linear variables.

To investigate the model fit, we followed the recommendations by Hosmer Lemeshow, 2013. Specifically, we manually inspected percentiles based on 10 grouped expected observed tables. We calculated (1) the Hosmed Lemeshow fit statistic [37], where *p* > 0.05 indicated a good fit, and (2) the area under the Receiver Operating Characteristic (ROC) curve (AUC) [36], where AUC = 0.70–0.80 indicated a fair fit and 0.80–0.90 indicated a good fit. Statistics were calculated using Stata version 18.0 for Windows [38].

#### 2.3.3. Attrition Analysis

There were a high number of missing values, in particular regarding the follow-up measures (neck pain intensity and neck-related disability), but also regarding the illness perceptions at the three-month follow-up. We decided to refrain from conducting multiple imputation as imputation is required separately in the exposure groups, and there is the problem of imputing both outcome and moderation variables. However, we performed an attrition analysis where individuals were categorised as missing if they were missing on any of the three variables with many missings (illness perceptions at the three-month follow-up, neck pain and neck-related disability at the one-year follow-up). Individuals without missings and individuals with missings on any of the three variables were crudely compared (categorical variables) using the Pearson’s χ^2^ test. Differences between individuals without missings and individuals with missings with regard to continuous variables (age and illness perceptions) were analysed using the Mann Whitney’s U test.

## 3. Results

### 3.1. Participant Characteristics

**Figure 2:**
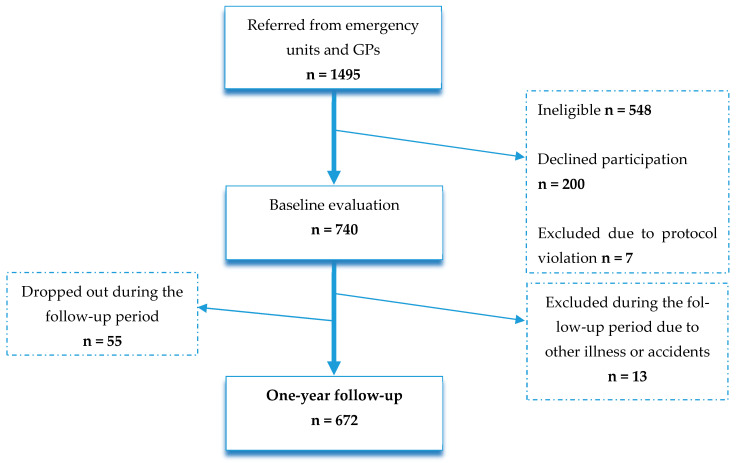
Flow chart describing inclusion and exclusion.

A total of 1495 participants were assessed for eligibility (Figure 2). Among these, 200 declined, seven were excluded due to protocol violations, and 548 were ineligible (22.6% of the ineligible participants could not be examined within ten days after the collision and 17.7% had injuries other than the whiplash trauma), leaving 740 for inclusion. The baseline questionnaire on illness perceptions was completed by 721 participants (97.4%), and three-month illness perceptions were completed by 534 participants (72.2%). The one-year follow-up questionnaires were completed by 672 participants, of whom 529 responded with the outcome of neck pain (overall response rate: 78.7%), and 514 responded with the outcome of neck-related disability (overall response rate: 76.5%). In total, 64.1% of the sample were women and the mean age of the participants was 34.8 years of age. Further demographic characteristics are presented in Table 1.

### 3.2. Multiple Logistic Regression Analyses

In the twelve models, we did not find a significant moderating effect of illness perceptions associated with receiving the three types of welfare benefits pre-collision or neck pain intensity and neck-related disability one-year post-collision (Table 2 and Table 3). However, we could see a trend towards illness perceptions at baseline and at the three-month follow-up having a moderating effect on the relationship between long-term sick leave and neck pain one year after the whiplash collision (OR (±95% CI) = 4.2 (0.91; 18.9) and (OR (±95% CI) = 3.2 (0.81; 12.6), respectively). According to Zielinsky et al. [34] both ORs are considered medium. The same consistent trend was not seen for neck-related disability. To illustrate the magnitude of the moderation, model estimates imply that comparing two persons both with a mean illness perception of 0 at the three-month follow-up, but only one of whom having experienced long-term sick leave before the accident, the person having experienced long-term sick leave would have a 130% greater odds of considerable neck pain one year after the accident (OR (±95% CI) = 2.3 (0.92; 5.6). Furthermore, if the person having experienced long-term sick leave had a mean illness perception of 1 at the three-month follow-up, the odds of considerable neck pain one year after the accident would be 2810% greater (OR (±95% CI) = 29.1 (10.3; 82.1). All models fall within a good model fit using the Hosmer-Lemeshow fit statistic, a fair-to-good model fit using the AUC, and a good-to-very good fit using a heuristic shrinkage estimate (Table 2 and Table 3).

### 3.3. Attrition Analysis

A high number of missing values were noted on the follow-up measures: neck pain intensity N = 211 (28.5%), neck-related disability N = 226 (30.5%), and illness perceptions at the three-month follow-up N = 206 (27.8%). We performed an attrition analysis of these variables, which is presented in Table 4. Individuals that were more likely to have missing data on either one of the two outcome measures or on illness perceptions at the three-month follow-up were men, younger, unskilled, receiving social assistance, and being short-term unemployed before the collision. There were no differences between the missings and non-missings regarding illness perceptions at baseline or sick leave before the collision.

## 4. Discussion

### 4.1. Summary of Findings

In this prospective study, we tested whether illness perceptions obtained a few days and three months after acute whiplash trauma moderated the association with three types of previously received welfare benefits and persistent neck pain and neck-related disability one year post-collision.

We did not find a significant moderating effect of illness perceptions. However, we could see a trend towards illness perceptions (at both baseline and at the three-month follow-up) having a moderating effect on the relationship between pre-trauma long-term sick leave and neck pain one year after the whiplash collision. We expected that the potential moderating effect of illness perceptions would increase between the baseline and the three months post-collision, yet we did not find that.

### 4.2. Interpretation of Findings

A systematic review showed that receiving welfare benefits was associated with negative illness perceptions [22]. In some cases, pre-collision welfare benefits were associated with poor recovery after whiplash trauma [12,13]. Further, negative illness perceptions have been shown to be associated with poor outcomes in whiplash and other conditions characterised by pain [17,23]. Still, no previous studies to our knowledge have investigated the moderating effect of early illness perceptions on the association between previously received welfare benefits and poor outcomes in WAD or other populations suffering with pain. We did not, however, find a statistically significant effect supporting our hypothesis regarding moderation in this study. The results, however, point in the expected direction regarding a moderating effect of illness perceptions on long-term sick leave, but not for social assistance welfare or unemployment. This would be expected, as one could receive social assistance for many reasons (e.g., not prone to work, marginalised people), and unemployment before the collision could be due to many reasons, such as a low labour demand in specific sectors. However, long-term sick leave before the collision is a health-related vulnerability that may affect a future health problem to a higher degree than social exclusion (social assistance and unemployment). With these considerations and our findings in mind, we would state that, regarding social assistance and unemployment, there may be no moderating effect related to illness perceptions, but regarding long-term sick leave, we might overlook a moderating effect. However, this would be considered a medium effect according to the interval cut offs suggested by Zielinski et al. [34]. Further research on this potential moderating effect is recommended, particularly regarding long-term sick leave before the collision. Firstly, by investigating a larger sample of patients that have sustained whiplash trauma. Our sample is large (N = 740), however long-term sick leave before the collision only applies to 19% of our cohort and when the potential moderator and the end points have missings, the cohort would have to be even larger. Secondly, other groups of patients with disability due to musculoskeletal pain could also be investigated regarding the moderation trend of long-term sick leave to examine whether this is specifically the case in whiplash trauma or applies to musculoskeletal pain in general. Studies would still have to include patients in which the onset of acute pain is specific, e.g., trauma-related, and where it is possible to measure the illness perceptions after the onset of acute pain.

The mechanisms involved in poor recovery after an acute whiplash trauma are multifactorial [8,9]. Factors other than illness beliefs may be more important for the association between previously received welfare benefits and later neck pain intensity or neck-related disability than illness perceptions, e.g., behavioural processes such as adaptive active pain coping, which has been found to decrease pain disability in chronic pain conditions [39]. Other factors before the collision may also prove essential, e.g., earlier illness perceptions or experience with the health system. Further, certain factors at the time of an accident have been found to negatively influence patients with musculoskeletal pain in the transition from acute to chronic pain [40], and factors such as high self-efficacy have been found to be a protective psychological resource in patients with persistent pain and a resilience factor associated with improved functional outcomes among patients with chronic pain [41]. As such, these may also be of importance in explaining poor recovery in WAD.

If negative illness perceptions actually moderate the association between earlier long-term sick leave and future pain and disability following whiplash trauma, then the clinical relevance would be noteworthy. Recommendations could then focus on clinicians’ devoting attention to the patient’s possible negative illness perceptions soon after the accident, e.g., via an early questionnaire, to detect such perceptions. In the case of both negative illness perceptions and previous long-term sick leave an intervention may be put in place. This intervention could be based on the common-sense model of illness [15] and, in the light end of the spectrum, psychoeducation on the relationship between previous life experiences of illness and pain and future negative illness perceptions, and the maladaptive coping behaviours that are sometimes a consequence of this connection. It may be enough for some patients to gain insight into these connections to be able to be more flexible in their coping behaviours. For those patients with more entrenched illness perceptions, a longer intervention including treatment-related improvements in cognitive content as recommended by Edwards et al. may be efficient [39].

### 4.3. Strengths and Limitations

This study is the first to examine the possible moderating effect of illness perceptions on the association between previously received welfare benefits and outcomes one year post-collision in WAD. A strength of this study is the relatively large sample size. Further, the study included acute whiplash patients referred consecutively from emergency units and general practitioners promptly post-trauma as opposed to some studies that recruit patients via insurance companies [42]. The latter studies may possibly include patients where litigation issues could introduce bias, and patients’ symptoms may have become chronic at the time of inclusion. On the other hand, recruiting patients from general practitioners and emergency units may also introduce bias. For instance, patients who seek emergency care might have more severe symptoms or different expectations and beliefs about whiplash and neck pain than patients who do not seek care at all or who would rather seek other types of care than emergency care. In Denmark, public health care services are free of charge for the patient. This makes access to emergency units and general practitioners easy, and obvious selection bias due to socio-demographic characteristics is avoided. However, there may still be a bias in terms of who agrees to participate in research studies. Further, participants were not included in this study if they had no symptoms of acute whiplash. This created a possible bias towards this sample having more symptoms and perhaps more pessimistic illness perceptions than the entire population of people experiencing acute whiplash trauma after car collisions. Another strength of the study is its prospective design. Furthermore, the information on the weeks of welfare benefits is drawn from registers, which provides a reliable measure as the information is not self-reported. Adding up the weeks of welfare benefits five years preceding the collision would not be reliable data if self-reported. Also, register data are complete, thus avoiding missing information. Our study further included a priori theory-based DAG assessment for confounding variables established with guidance from an expert group, including a comprehensive list of potential confounders.

However, there are also several limitations. One limitation might be that we have only self-reported outcome measures. However, WAD is not determined by objectively defined disease markers, and therefore the use of subjective ratings is unavoidable. To deal with this, participants not only reported neck pain intensity but also neck-related disability to include a measure of functional ability. Using two different outcome measures offers the opportunity of more viable results. Another limitation regarding the outcome measures is the dichotomisation, e.g., a score of 4 for neck pain is considered equal to a score of 9. However, the dichotomisation was chosen as the best possible option for the data, as the observations were not linear and they were skewed, i.e., we have numerous observations with 0. Furthermore, we may have a potential lack of power which is indicated by the rather wide confidence intervals in this study. It is a limitation that this study on illness perceptions, welfare benefits, and recovery did not include a sample size analysis previous to the study. This study is a secondary analysis on a study that was originally designed to include two RCT studies, and at that time a sample size analysis was performed but for the purpose of the RCTs and unfortunately not for this current study.

Our attrition analysis showed that the group that had missing data on either the outcome measures or illness perceptions at the three-month follow-up were more likely to be men, younger, unskilled, or receiving social assistance or short-term unemployment benefit before the collision. If these groups (that are more likely to be missing) have a different interaction effect, it could lead to bias. Although no differences in illness perceptions were found at baseline, the missing data at the three-month follow-up could indicate unobserved differences in perceptions over time between those with and without complete data. If illness perceptions change differently for the groups with missing data, this could affect the validity of the interaction effect in our analysis. Moreover, the bias in the analysis of the interaction effect can reduce the generalisability of our results, i.e., the findings might not apply to the entire population but rather only to those who were more likely to have complete data (women, older, with further education, receiving no social assistance and having no unemployment before the collision). However, since there is no difference between the missing and non-missing groups regarding long-term sick leave, the missing data is less likely to introduce bias in the estimation of the interaction effect for this specific welfare benefit. This suggests that the trend towards an interaction effect observed for long-term sick leave is more reliable and less likely to be overestimated due to missing data. The lack of systematic missingness related to this variable means that our findings for a trend towards an interaction effect with illness perceptions should be more robust. Furthermore, the lack of interaction effects for unemployment and social assistance is likely unaffected by the missing data, and the attrition analysis reinforces the validity of these non-findings. Therefore, we can be more confident that the trend towards the interaction effect of long-term sick leave is not an overestimation, while the absence of interaction effects for the other benefits is also a reliable conclusion.

Regarding the generalisability of the results to other countries besides Denmark, we believe that the findings can be applied to other societies as many societies have welfare benefits, some provided by the employer and some by the government. In particular, sick leave benefit is well-known and widely used, whereas social assistance benefit and unemployment benefit are not as well-established worldwide. Regardless of who provides the welfare benefits or whether such benefits are available, it remains important from a clinical perspective to focus on the subgroups of patients who have been ill, unemployed, or in other ways socially excluded prior to experiencing whiplash trauma.

## 5. Conclusions

In this study, we did not find a consistent moderating effect of welfare benefits before the collision on future increased neck pain intensity and neck-related disability. Replicating the results of this study is necessary to further investigate whether illness perceptions moderate the association between earlier received welfare benefits and later poor recovery in other populations. Large interaction effects of illness perceptions on long-term sick leave could have been overlooked due to methodological matters. Although our study did not find a significant moderating effect, our results indicate the potential importance of illness perceptions in the complex recovery process, with strong direct associations in the current study. If many different factors are important in the recovery process, identifying the moderation effects on specific factors can be difficult.

## Figures and Tables

**Table 1:** Characteristics of overall sample regarding socio-demographic variables, welfare benefits, illness perceptions, and outcome.

		Overall Sample
Pre-collision measures five years back		N = 740
Social assistance, N (%)	1: No social assistance	608 (82.2)
	2: >=1 week Missing data, N = 0	132 (17.8)

		
Sick leave, N (%)	1: No sick leave	423 (57.2)
	2: Short term sick leave (1–12 weeks)	178 (24.1)
	3: Long term sick leave (>12 weeks) Missing data, N = 0	139 (18.8)
		
Unemployment, N (%)	1: No unemployment	409 (55.3)
	2: Short term unemployment (1–52 weeks)	204 (27.6)
	3: Long term unemployment (>52 weeks) Missing data, N = 0	127 (17.2)
		
Baseline measures		
Sex, N (%)	Female	474 (64.1)
	Missing data, N = 0	
Education (vocational), N (%)	Unskilled	150 (20.3)
	Skilled	231 (31.2)
	Further education <= 4 years Further education > 4 years	167 (22.6)
		63 (8.5)
	Other	83 (11.2)
	Missing data	46 (6.2)
Negative illness perceptions, mean (SD)	Range (0–3) Missing data, N = 19 (2.6%)	0.49 (0.52)

Age years, mean (SD)	Range 18–67 years	34.8 (11.4)
	Missing data, N = 0	
		
Post-collision measures three-months follow-up		
Negative illness perceptions, mean (SD)	Range 0–3	0.59 (0.68)
	Missing data N = 206 (27.8%)	
		
Outcome measures one-year follow-up		
Considerable neck pain, N (%)	Yes	189 (25.5)
	Missing data	211 (28.5)
Considerable neck-related disability, N (%)	Yes	179 (24.2)
	Missing data	226 (30.5)

SD = standard deviation.

**Table 2:** Multivariate model with odds ratios of potential interactions between receiving welfare benefits before the collision and illness perceptions at baseline on neck pain and neck disability at the one-year follow-up.

	Neck Pain ^a,b,i^		Neck-Related Disability ^c,d,j^	
Social Assistance	OR (±95% CI)	*p* Value	OR (±95% CI)	*p* Value
1. Social assistance before collision (≥1 week)	1.6 (0.73; 3.6)	0.242	**2.5 (1.1; 5.5)**	**0.029**
2. Negative illness perceptions at baseline (mean)	**3.4 (2.1; 5.5)**	**<0.001**	**4.0 (2.4; 6.5)**	**<0.001**
3. Interaction (illness perceptions x social assistance)				
Social assistance (≥1 week)	0.72 (0.25; 2.1)	0.535	0.55 (0.18; 1.6)	0.278
	Neck pain ^a,e,i^		Neck-related disability ^c,f,j^	
Sick leave	OR (±95% CI)	*p* value	OR (±95% CI)	*p* value
1. No sick leave before collision Short term sick leave (1–12 weeks)	0.94 (0.44; 2.0)	0.862	0.99 (0.46; 2.1)	0.986
Long term sick leave (>12 weeks)	**2.5 (1.1; 5.8)**	**0.029**	**2.6 (1.1; 6.2)**	**0.029**
2. Negative illness perceptions at baseline (mean)	**2.2 (1.2; 3.8)**	**0.006**	**2.8 (1.6; 4.8)**	**<0.001**
3. Interaction (illness perceptions × sick leave)				
Short term sick leave (1–12 weeks) Long term sick leave (>12 weeks)	1.9 (0.64; 5.8) 4.2 (0.91; 18.9)	0.242 0.066	1.5 (0.49; 4.5) 3.0 (0.64; 14.2)	0.480 0.163
	Neck pain ^a,g,i^		Neck-related disability ^c,h,j^	
Unemployment	OR (±95% CI)	*p* value	OR (±95% CI)	*p* value
1. No unemployment before collision				
Short term unemployment (1–52 weeks)	1.0 (0.51; 2.1)	0.930	0.84 (0.40; 1.8)	0.634
Long term unemployment (>52 weeks)	1.2 (0.53; 2.5)	0.716	1.3 (0.58; 2.9)	0.527
2. Negative illness perceptions at baseline (mean)	**2.8 (1.6; 5.1)**	**<0.001**	**3.4 (1.9; 6.2)**	**<0.001**
3. Interaction (illness perceptions × unemployment)				
Short term unemployment (1–52 weeks)	1.0 (0.39; 2.7)	0.959	1.1 (0.40; 3.1)	0.846
Long term unemployment (>52 weeks)	2.2 (0.59; 7.9)	0.243	1.2 (0.34; 4.8)	0.710

^a^ Considerable neck pain (*n* = 170, 35%) compared to minimal neck pain (n = 318, 65%). ^b^ Hosmer-Lemeshow fit statistic χ^2^(8) = 3.63, *p* = 0.89. The area under the ROC curve (AUC) 0.70. Heuristic shrinkage estimate 0.84. ^c^ Considerable neck-related disability (n = 166, 35%) compared to minimal neck-related disability (n = 310, 65%). ^d^ Hosmer-Lemeshow fit statistic χ^2^(8) = 2.44, *p* = 0.96. The area under the ROC curve (AUC) 0.71. Heuristic shrinkage estimate 0.86. ^e^ Hosmer-Lemeshow fit statistic χ^2^(8) = 2.28, *p* = 0.97. The area under the ROC curve (AUC) 0.75. Heuristic shrinkage estimate 0.88. ^f^ Hosmer-Lemeshow fit statistic χ^2^(8) = 3.26, *p* = 0.92. The area under the ROC curve (AUC) 0.73. Heuristic shrinkage estimate 0.88. ^g^ Hosmer-Lemeshow fit statistic χ^2^(8) = 4.55, *p* = 0.80. The area under the ROC curve (AUC) 0.70. Heuristic shrinkage estimate 0.82. ^h^ Hosmer-Lemeshow fit statistic χ^2^(8) = 2.88, *p* = 0.94. The area under the ROC curve (AUC) 0.70. Heuristic shrinkage estimate 0.82. ^i^ N = 488. ^j^ N = 476. Bold estimates = significant results.

**Table 3:** Multivariate model with odds ratios of interactions between receiving welfare benefits before the collision and illness perceptions at the three-month follow-up on neck pain and neck disability at the one-year follow-up.

	Neck Pain ^a,b,i^		Neck-Related Disability ^c,d,j^	
Social Assistance	OR (±95% CI)	*p* Value	OR (±95% CI)	*p* Value
1. Social assistance before collision (≥1 week)	1.8 (0.73; 4.4)	0.202	1.9 (0.78; 4.8)	0.158
2. Negative illness perceptions at 3-months FU (mean)	**6.1 (3.8; 9.6)**	**<0.001**	**6.5 (4.0; 10.5)**	**<0.001**
3. Interaction (illness perceptions × social assistance)				
Social assistance (≥1 week)	0.52 (0.21; 1.3)	0.176	0.69 (0.24; 2.0)	0.494
	Neck pain ^a,e,i^		Neck-related disability ^c,f,j^	
Sick leave	OR (±95% CI)	*p* value	OR (±95% CI)	*p* value
1. No sick leave before collision Short term sick leave (1–12 weeks)				
	0.92 (0.39; 2.2)	0.853	1.1 (0.45; 2.7)	0.830
Long term sick leave (>12 weeks)	2.3 (0.92; 5.6)	0.074	**6.0 (2.5; 14.5)**	**<0.001**
2. Negative illness perceptions at 3-month FU (mean)	**4.0 (2.2; 7.1)**	**<0.001**	**7.6 (3.9; 14.6)**	**<0.001**
3. Interaction (illness perceptions × sick leave)				
Short term sick leave (1–12 weeks) Long term sick leave (>12 weeks)	1.2 (0.46; 3.0) 3.2 (0.81; 12.6)	0.727 0.094	0.71 (0.25; 2.1) 0.54 (0.18; 1.6)	0.530 0.278
	Neck pain ^a,g,i^		Neck-related disability ^c,h,j^	
Unemployment	OR (±95% CI)	*p* value	OR (±95% CI)	*p* value
1. No unemployment before collision				
Short term unemployment (1–52 weeks)	1.7 (0.75; 3.8)	0.205	0.76 (0.31; 1.8)	0.545
Long term unemployment (>52 weeks)	1.1 (0.45; 2.8)	0.809	1.1 (0.47; 2.7)	0.785
2. Negative illness perceptions at 3-month FU (mean)	**6.1 (3.4; 10.8)**	**<0.001**	**6.1 (3.3; 11.1)**	**<0.001**
3. Interaction (illness perceptions × unemployment)				
Short term unemployment (1–52 weeks)	0.66 (0.26; 1.7)	0.384	1.3 (0.46; 4.0)	0.591
Long term unemployment (>52 weeks)	0.91 (0.34; 2.4)	0.842	0.78 (0.28; 2.2)	0.640

^a^ Considerable neck pain (n = 149, 35%) compared to minimal neck pain (n = 277, 65%). ^b^ Hosmer-Lemeshow fit statistic χ^2^(8) = 5.40, *p* = 0.71. The area under the ROC curve (AUC) 0.79. Heuristic shrinkage estimate 0.92. ^c^ Considerable neck-related disability (n = 153, 37%) compared to minimal neck-related disability (n = 264, 63%). ^d^ Hosmer-Lemeshow fit statistic χ^2^(8) = 9.39, *p* = 0.31. The area under the ROC curve (AUC) 0.81. Heuristic shrinkage estimate 0.93. ^e^ Hosmer-Lemeshow fit statistic χ^2^(8) = 11.8, *p* = 0.16. The area under the ROC curve (AUC) 0.81. Heuristic shrinkage estimate 0.92. ^f^ Hosmer-Lemeshow fit statistic χ^2^(8) = 3.00, *p* = 0.93. The area under the ROC curve (AUC) 0.83. Heuristic shrinkage estimate 0.92. ^g^ Hosmer-Lemeshow fit statistic χ^2^(8) = 8.81, *p* = 0.36. The area under the ROC curve (AUC) 0.79. Heuristic shrinkage estimate 0.90. ^h^ Hosmer-Lemeshow fit statistic χ^2^(8) = 5.33, *p* = 0.72. The area under the ROC curve (AUC) 0.81. Heuristic shrinkage estimate 0.91. ^i^ N = 426. ^j^ N = 417. Bold estimates = significant results. FU = Follow-up.

**Table 4:** Attrition analysis on illness perceptions at three-months follow-up and the two outcome measures.

Pre-Collision Measures Five-Years Back		Non-Missing on the Three Measures N = 444 (60%)	Missing on at Least One of the Three Measures * N = 296 (40%)	χ^2^ Test		
				χ^2^	*df*	*p*
Social assistance, N (%)	1: No social assistance	383 (86.3)	225 (76.0)			
	2: ≥1 week Missing data, N = 0	61 (13.7)	71 (24.0)	12.7	1	<0.001
		
				
Sick leave, N (%)	1: No sick leave	269 (60.6)	154 (52.0)			
	2: Short term sick leave (1–12 weeks)	99 (22.3)	79 (26.7)			
	3: Long term sick leave (>12 weeks) Missing data, N = 0	76 (17.1)	63 (21.3)	5.3	2	0.069
				
Unemployment, N (%)	1: No unemployment	260 (58.6)	149 (50.3)			
	2: Short term unemployment (1–52 weeks)	106 (23.9)	98 (33.1)			
	3: Long term unemployment (>52 weeks) Missing data, N = 0	78 (17.6)	49 (16.6)	7.8	2	0.021
				Mann Whitney’s U test		
Baseline measures				U	z	*p*
Age years, mean (SD)	Missing data N = 0	36.2 (11.8)	32.7 (10.6)	8,115,428.00	3.9	**<0.0001**
Negative illness perceptions at baseline, mean (SD)	Missing data N = 19	0.46 (0.48)	0.54 (0.58)	7,372,178.33	−0.765	0.4445
				χ^2^ test		
				χ^2^	*df*	*p*
Sex, N (%)	Female	304 (68.5)	170 (57.4)			
	Male Missing data N = 0	140 (31.5)	126 (42.6)	9.4	1	**0.002**
Education (vocational), N (%)	Unskilled	75 (18.1)	75 (26.8)			
	Skilled	141 (34.1)	90 (32.1)			
	Further education ≤ 4 years Further education > 4 years	114 (27.5)	53 (18.9)			
		40 (9.7)	23 (8.2)			
	Other	44 (10.6)	39 (13.9)	13.0	4	**0.011**
	Missing data N = 46					

* Negative illness perceptions at 3-month follow-up, neck pain, neck-related disability at 12-month follow-up. SD = standard deviation. df = degrees of freedom. Bold *p*-values = significant.

## Data Availability

The data presented in this study are available on request from the corresponding author due to data still being analysed in different studies.
